# Unveiling evolutionary cradles and museums of flowering plants in a neotropical biodiversity hotspot

**DOI:** 10.1098/rsos.230917

**Published:** 2023-10-11

**Authors:** Carlos E. González-Orozco

**Affiliations:** Corporación Colombiana de Investigación Agropecuaria- Agrosavia, Centro de Investigación La Libertad- Km 14 vía Villavicencio-Puerto López, Meta, Colombia

**Keywords:** neoendemism, palaeoendemism, flora, northern Andes, spatial phylogenetics, Colombia

## Abstract

Colombia, renowned as an important centre of global biodiversity, continues to harbour undiscovered evolutionary hotspots of flowering plants. The altitude-dependent hypothesis suggests that richness patterns are determined by altitude and probably influenced by climate variables. This study employs null models based on a species-level phylogeny of Colombia's flowering plants and their geographical distributions to identify evolutionary hotspots. We explore the potential correlation between elevation, climate variables such as temperature and rainfall, and the location and nature of these hotspots. The findings reveal that evolutionary cradles, which house young endemic species, are predominantly located in the mountainous regions of the Andes. Conversely, evolutionary museums, hosting older endemic species, are found in lowland regions spanning the Caribbean, Orinoco, Amazon and Pacific areas. These results demonstrate a clear elevational segregation of evolutionary hotspots, primarily influenced by temperature, thereby supporting the hypothesis under examination. Furthermore, this study identifies previously unrecognized evolutionary regions, highlighting the limited understanding of Colombia's biodiversity distribution and evolutionary history.

## 1. Introduction

Hotspots of biodiversity, characterized by a concentration of recently derived species, are commonly referred to as ‘cradles’, whereas areas with a high concentration of early-divergent species are known as ‘museums’ of biodiversity [1,2]. These conceptual frameworks initiated three decades ago. A pioneering study in this field focused on discovering relicts of old and areas of young species of birds in Africa and South America [3]. Through geographical patterns, this illustrated how birds exhibited diverse evolutionary origins, influenced by their specific environments. For example, evolutionary fronts—locations hosting recently evolved species—are prominent in regions of active speciation, such as the Andes. By contrast, lowland rainforests serve as reservoirs for relict populations characterized by older origins. The results also showed that range-restricted species found at higher elevations, which are potential cradles, exhibited elevated speciation rates alongside lower species richness—potentially due to heightened rates of extinction. These patterns might be related to mountains geographical features, or elevation, which play a key role in speciation [4,5].

Although a solid conceptual baseline and hypothesis already exist [6], their application require evaluation through alternative methodological approaches across various biological groups and latitudes. For instance, some of the new frontiers in genetics offer an exciting opportunity to explore such concepts using evolutionary models. One of the latest examples identified hummingbird cradles and museums in the northern Andes [7]. They found that the cradles of endemic species are situated in high-altitude mountain areas, while lowland or midland forest areas harbour the museum-type species, which belong to an older evolutionary origin.

This study uses the evolutionary relationships among branches of the tree of life, a concept known as phylogenetic diversity (PD) [8]. It is relevant to bear in mind that despite evolutionary metrics adding new levels of information, species-based hypothesis can be comparable to PD because it has been demonstrated that species richness and PD are strongly correlated [9]. However, PD can provide estimates based on evolutionary relationships that species alone cannot [10]. Given that the phylogenetic approach delves deeper into understanding biodiversity, it is anticipated that PD approaches will enhance our current comprehension of biodiversity. For instance, the emergence of approaches such as ‘spatial phylogenetics' [11] amplifies analytical capabilities by incorporating evolutionary diversity metrics derived from PD. These approaches contribute to identifying centres of palaeo and neoendemism, considering the evolutionary relationships among closely or distantly related species across the entire tree of life. These types of centres can be considered as cradles or museums of biodiversity [12–14].

In terms of plant evolutionary diversity, Colombia remains largely unexplored, offering ample opportunities for new discoveries [15]. However, it is an ideal country for testing ecological hypotheses due to its highly complex geography, characterized by the mountain ranges, abundant biota, rough topography and diverse evolutionary history, which give rise to numerous types of ecosystems within the same region. The northern Andes region, to which Colombia belongs, further contributes to its uniqueness, as no other region in the Neotropics possesses such distinctive features that host many narrowly distributed species proving to be unique organisms [16]. Particularly noteworthy is the presence of three Andean Cordilleras that stretch for 1200 km, with elevations ranging from sea level to perpetual snow close to 6000 m above sea level. This remarkable geographical characteristic fosters the development of a highly diverse biota within the equatorial zone of the Neotropics, recognized as a biodiversity hotspot.

The northern Andes have been considered an area capable of harbouring both cradles and museums of biodiversity [1,5]. For example, in the case of tropical hummingbird species, research has revealed that the Andean mountain ranges of Colombia contain localized areas with endemic species of both recent (young) and ancient (old) origin [7]. In fact, the Sierra Nevada de Santa Marta, which is an isolated massif, represents the clearest example of a cradle for young endemic species in Colombia, while the western mountain range displays more characteristics of a museum for endemic hummingbird species [7]. Consequently, this shows that the species' geographical range size also emerges as a crucial factor in explaining diversity patterns in the northern Andes. Even within the Amazon region, studies on phylogenetic diversity of tree species at the community level across the different Amazon biomes have demonstrated that species geographical ranges changes over space and are influenced by the type of geological substrate [17].

Unfortunately, the spatial patterns of evolutionary diversity in Colombia's flowering plants remain undefined, which leaves fundamental questions about their distribution unanswered. It is of utmost importance to untangle the spatial distribution of these centres and ascertain whether they overlap or remain separate in order to investigate the underlying patterns and processes that shape biodiversity. One hypothesis proposes that the persistence of new species is influenced by stability of their habitat. For example, it posits that regions of high biodiversity might coexist within ecologically stable areas in the tropical Andes region [3]. Another prominent hypothesis suggests that species richness might be linked to elevational changes, wherein specific climate zones develop, and land area decreases as one ascends the mountain slopes [4].

Clarifying the variables that dictate the distribution of plants in Colombia represents a significant challenge. Although numerous hypotheses have been proposed, very few have been rigorously tested. For instance, research on the distribution of neotropical birds in the Andes of Peru has shown a correlation between elevation and the speciation process [2]. This insight supports the notion that diversification in tropical regions tends to intensify at higher altitudes. In a different scenario, diversity could also be influenced by environmental factors associated with elevation gradients. Considerable the substantial variation in elevational ranges across Colombia, climate variables, including precipitation and temperature, are likely to play a crucial role in explaining the biogeography and distribution patterns of plant biodiversity throughout the country. Notably, it is recognized that for every 100 m increase in elevation along the Andean Mountain ranges in Colombia, there is an approximate 1° of temperature decrease. Considering these hypotheses, temperature can be regarded as a dependent variable, while precipitation may exert an independent influence on plant distributions.

Here, the aim is to identify cradles and museums of flowering plants in Colombia and test the altitude-dependent hypotheses [4]. Spatial phylogenetics methods [11,12] were applied to a subset of the flowering plants from Colombia, and data on average and extreme climate for rainfall and temperature. It was tested whether the distribution patterns of palaeoendemism centres overlap with neoendemism centres. Specifically, it was explored how low-altitude regions harbour evolutionary hotspots characterized by a concentration of range-restricted long branches, which can be considered ‘centres of palaeoendemism’ or museums of ancient evolutionary diversity where old species survive. Conversely, high-altitude mountain regions contain evolutionary hotspots with a concentration of range-restricted short branches, which can be considered ‘centres of neoendemism’ or cradles of recently diverged species where new species arise.

To examine the relationship between climate data and areas of palaeo and neoendemism, it was explored whether evolutionary hotspots would exhibit distinct responses to changes in climate variables based on contrasting altitudes. Specifically, it was expected that hotter lowland conditions would correspond to areas of palaeoendemism, while cooler mountainous conditions would correspond to areas of neoendemism. Therefore, the study was guided by the hypothesis that altitude, and consequently the associated climate variables, exert a significant causal effect on the spatial distribution patterns of evolutionary hotspots within the studied plant groups.

Some examples of cradles and museums of biodiversity in South America can be found in the vascular flora of Chile or Mexico [18,19]. In the case of Chile, results revealed that centres of palaeoendemism are situated in southern Chile, while centres of neoendemism are found in the northern regions. Similarly, in the coastal zone of central Chile, recognized as a hotspot of flora diversity, areas displaying museum and cradle diversity were identified within regions rich in woody plants [20]. In Mexico, cradles and museums of plants were found in tropical dry forests [19].

Despite global advancements in biodiversity, the application of spatial phylogenetics within the tropics remains limited [6]. What is even more concerning is the absence of evolutionary-based research conducted thus far to identify the centres of palaeoendemism or neoendemism for flowering plants in neotropical countries like Colombia. This baseline information is urgently required to improve conservation of biodiversity in equatorial latitudes. To address this gap, the Biodiverse v. 4.3 software [21] was employed for our analysis (see Material and methods for details). Based on a previous dataset [22], occurrence data for flowering plants in Colombia were carefully extracted and mapped at a scale of 0.1° × 0.1° (approx. 10 km). By pruning a global species-level phylogeny of angiosperm plants [23], we constructed a tree that specifically matched the species found in Colombia. This yielded a comprehensive dataset comprising 20 342 records, representing 2876 branches of the phylogenetic tree. Leveraging this pruned phylogeny, we conducted spatial phylogenetic analysis, specifically focusing on three indices derived from phylogenetic diversity. The first index, relative phylogenetic diversity (RPD), quantified the proportion of branches present at each site, while the second index, relative phylogenetic endemism (RPE), calculated the phylogenetic diversity in relation to the species' distribution ranges [12]. Significance was assigned using two-tailed tests as both extremes of the distribution were of statistical interest. Both RPD and RPE yield a distribution that can have high values (greater than 0.99) or low values of significance (less than 0.01). Areas of significantly high RPE were considered as centres of palaeoendemism, which are proposed here as cradles. On the contrary, areas of significantly low RPE were considered as centres of endemism, which are proposed as museums.

Additionally, a phylogenetic clustering analysis known as range weighted phylogenetic endemism was conducted to assess the degree of uniquely shared evolutionary diversity among the different biogeographic regions of Colombia. These comprehensive analyses not only enabled us to identify cradle (neoendemism) and museum areas (palaeoendemism), but also provided insights into potential connectivity and interrelations among the various bioregions. Furthermore, by employing these three indices, the altitude-dependent hypothesis was tested.

## 2. Material and methods

### 2.1. Species distributions

The geographical distributions of the analysed flowering plant species were obtained from a previously published database [22]. This original database comprises 271 578 georeferenced records representing 20 342 species. However, the analysed species dataset matching the phylogeny contains 2875 species corresponding to 1368 genera of flowering plants. Digitizing the biological diversity of Colombia, the second most biodiverse country in the world, poses a significant challenge. Consequently, the spatial data of plants in Colombia may not fully represent a large percentage of the existing flora and its actual coverage [24]. Although the current sampling does not yet reach the international standards, open-access flora data for Colombia, such as the Global Biodiversity Information Facility (GBIF) or the Botanical Information and Ecology Network (BIEN), can be considered acceptable despite the sampling biases.

In the initially published plant distribution database for Colombia [22], it was observed that the biogeographic patterns were successfully captured, instilling confidence that future analyses would accurately reflect the spatial patterns of biodiversity within each biogeographic province, and even more robust by adding the evolutionary facet. To provide readers with an understanding of potential spatial biases and to acknowledge the limitations of available databases for Colombia, an index called sampling redundancy was employed to evaluate the sampling strength (see electronic supplementary material, figure S1).

### 2.2. Phylogenetic data

The phylogenetic tree used here was extracted from a previously published dated phylogeny [23]. The phylogeny was designed for global scale and includes a total of 36 101 angiosperm species distributed across 8399 genera. The final topology results from maximum-likelihood tree inference with RAxML 7.4.2. The phylogeny was pruned at the level of Colombia using the previous species list [19]. As a result, a pruned phylogeny was generated, consisting of 2875 species or terminal branches of the tree. This phylogeny is freely accessible and available as a .tre file. This phylogeny was previously used in other studies for the same type of analysis, showing positive results [6].

### 2.3. Phylogenetic analysis

All spatial phylogenetic analyses were conducted using the Biodiverse v. 4.3 package [21]. The initial step involved mapping the distribution of species across mainland Colombia, which comprised 4218 grids of 0.1° × 0.1° size, approximately 1 km^2^ per grid. This fine spatial scale was selected to capture the specific characteristics of Colombia's rough geography. The spatial phylogenetic analysis employed three phylogenetic derived indices: relative phylogenetic diversity (RPD), relative phylogenetic endemism (RPE) [12], and range weighted phylogenetic turnover (RWT) [25]. These indices combined spatial distributions and phylogeny while employing null models to test for statistical significance.

We calculated the PD based on Faith's proposal [8]. RPD calculates the difference between PD measured on the original tree and PD measured on an alternative tree with the same number of nodes, where branch lengths are adjusted to be the same length. Similarly, RPE compares the difference between endemic phylogenetic diversity measured on the original tree and phylogenetic endemism (PE) measured on an alternative tree with the same number of nodes, where branch lengths are adjusted to be the same length. The PE metric was developed by Rosauer in 2009 [26].

Randomization tests, for RPD and RPE significance, were designed to test alternative hypotheses, considering that species can occur in the same locality [12]. These alternative tests measure them independently. Randomization tests were calculated 999 times for each grid for each metric. This allowed for multiple randomizations of distributions to generate an expected null distribution, which was compared against the observed distribution. Significance was assigned using two-tailed tests, as both extremes of the distribution were of statistical interest. Both RPD and RPE yield a distribution that can have high values (greater than 0.99) or low values of significance (less than 0.01). Areas of significantly high RPE were considered as centres of palaeoendemism, which are proposed here as cradles. On the contrary, areas of significantly low RPE were considered as centres of endemism, which are proposed as museums. The results of these spatial significance tests are automatically mapped and displayed in the visual tools of the Biodiverse software, generating maps of significance statistics for each index. Areas of Colombia that did not have species occurrences were not included in the calculations.

The last phylogenetic analysis was the RWT [25]. This index involves using the randomized PE, but this time it is calculated over biogeographic regions rather than individual grid cells. This provides an agglomerative and Euclidean distance matrix to map RWT. The result of this index is a map of the endemic phylogenetic regions, which is quite useful for investigating macroecological and biogeographic patterns of species. RWT was quantified at both 0.1° and 0.5° in grid cell sizes. The broader scale allows to identify more easily the evolutionary regions as they display more clearly than the fine resolution scale.

### 2.4. Elevational analysis

The RPD and RPE results were mapped in QGIS as grid-type files. They were then converted to shape files to overlay them with a 30 m resolution digital elevation model (DEM). Elevation values were extracted and assigned to each RPD and RPE grid. A standard boxplot and whiskers analysis was applied to the elevation differences between centres of palaeoendemism and neoendemism.

### 2.5. Climate analyses

To encompass a broader spectrum of environmental data across different spatial and temporal scales, both average and extreme climate data were analysed. Specifically, for the extreme climate analysis, maximum rainfall and temperature were used. The rasters had a spatial resolution of 3 × 3 km. These layers were generated by interpolating daily records spanning from 1980 to 2010, which were collected from 4438 weather stations in Colombia [27]. The interpolation of variables was carried out using the method REGNIE [28].

Regarding the average climate analysis, average rainfall and temperature variables were used. The rasters had a spatial resolution of 1 × 1 km. These rasters were derived from the macro climate dataset WordlClim v. 2 [29].

Using QGIS, the grid cells representing palaeoendemism and neoendemism were converted into points. Subsequently, the shapefile containing the occurrences of the evolutionary hotspots was overlaid onto the extreme and average rasters, enabling the extraction of the corresponding values for each location of palaeo and neoendemism.

After preparing the points and climate rasters, a statistical analysis was performed using GraphPad tool (https://www.graphpad.com). The analysis focused on exploring the relationship between the average and extreme climate variables and the evolutionary hotspots. The differences in average and extreme temperature and rainfall between the evolutionary hotspots were then analysed using a Student's *t*-test.

Lastly, a standard boxplot and whiskers analysis was applied to visualize and extract basic statistics between the climate variables and the centres of palaeoendemism and neoendemism. This analysis aimed to provide a comprehensive representation of the data and highlight any significant differences in the climate variables associated with the two evolutionary hotspots.

### 2.6. Species diversity analysis

As additional information, two species-based diversity metrics were calculated (see results in electronic supplementary material, figure S1). Species richness (SR) and corrected weighted endemism (CWE) were calculated in Biodiverse v. 4.3 software at the same grid size as the phylogenetic analyses (see electronic supplementary material, figure S2). CWE is the degree to which ranges of species found in a grid cell are restricted to that grid cell and SR refers to the number of species per grid cell [30,31]. A randomization test using the same method as explained above was applied to test the significance sites for CWE.

## 3. Results and discussion

### 3.1. Cradles and museums of biodiversity

**Figure 1.**
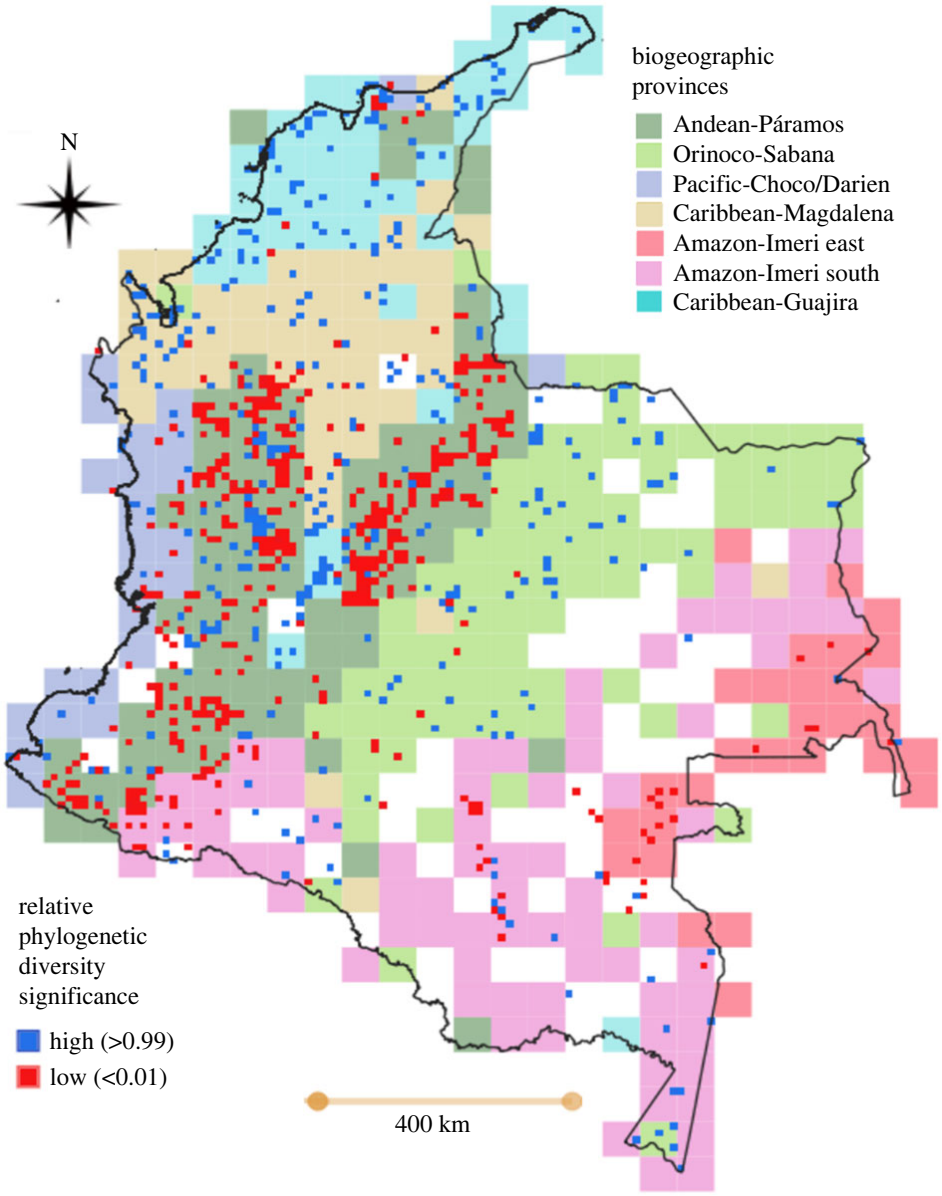
Spatial patterns of relative phylogenetic diversity of flowering plants in Colombia. Background map shows the Biogeographic provinces of Colombia according to Gonzalez-Orozco [19]. Significantly high values (greater than 0.99) indicate a concentration of long branches whereas significantly low values (less than 0.01) indicate a concentration of short branches.

We found a concentration of short branches in the Andean mountainous region. By contrast, the lowland regions, including the Amazon, Orinoco, Pacific, Caribbean and inter-Andean valleys showed a concentration of long branches (figure 1). The presence of long branches within an area may indicate that it served as a refuge, while the presence of short branches suggests areas of recent diversification as previously reported in the Neotropics [3,5,7].

**Figure 2.**
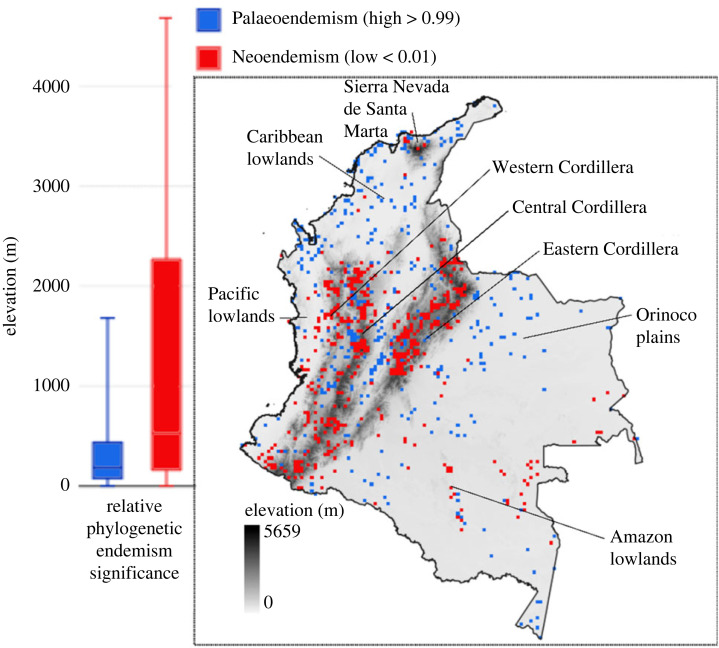
Cradles (neoendemism, *n* = 1044) and museums (palaeoendemism, *n* = 315) of flowering plants in Colombia. Significantly high values (greater than 0.99) indicate the presence of long-range restricted branches, or palaeoendemism, whereas significantly low values (less than 0.01) indicate the presence of short-range restricted branches or neoendemism.

To identify the spatial patterns of evolutionary cradles, known as ‘neoendemism’, and museums, referred to as centres of ‘palaeoendemism’, a null model that combined distribution ranges and phylogenetic diversity and endemism was employed (see Material and methods). The results revealed a clear geographical segregation of the centres of evolutionary diversity (figure 2), which contradicts the idea that diversity hotspots overlap as previously reported [7]. The analysis showed that museum areas were predominantly located in low elevations (median = 186 m; max = 1681 m), including the Caribbean dry and wet regions, Orinoco plains, Pacific forests, Amazon basin, and the Magdalena and Cauca inter-Andean valleys (figure 2). These areas were characterized by the presence of species with long range-restricted branches, indicating their status as centres of palaeoendemism. This finding is consistent with the notion that Amazonia is considered the main source of neotropical diversity [32].

On the other hand, areas of cradles are predominantly located in high mountainous regions of the Andes, as well as mid-elevations within the foothills of the Cordilleras and plateaus (median = 529 m; max = 4683 m) (figure 2). These areas indicated the presence of species with short range-restricted branches, suggesting their status as centres of neoendemism. This pattern may be associated with the biogeographical history of the region, potentially linked to the uplifting of the Andean Cordilleras [33]. Alternatively, the ecological characteristic may have influenced its distribution due to processes of evolutionary filtering or climate stability [34].

Interestingly, certain areas in the Amazon, such as outcrops, Sierras and the Serranías within the Choco biogeographic region, have been discovered to host both cradles and museums of biodiversity. These areas might have functioned as evolutionary refuges, offering unique ecological conditions that promoted species diversification and persistence. Another noteworthy example is the Sierra Nevada de Santa Marta in the Caribbean region, which represents a rare instance of mixed evolutionary centres. These findings, in conjunction with the centres of palaeo and neoendemism, align with the revelations made about neotropical birds [3]. Notably, the study observed that widely distributed species predominantly inhabit lowland rainforests, while areas with complex ecological conditions, such as mountain ranges, house new species that probably evolved within specific local environments. This observation stands as one of the pioneering instances of such patterns in the Neotropics of South America [3].

However, a significant distinction between the study on neotropical birds and the current paper lies in the fact that the former used branch length and its evolutionary relationships within the tree of life analysed under phylogenetic null models. Consequently, due to the methodological differences, an additional layer of evolutionary information has been introduced to further support the theory, as demonstrated in recent studies [7].

### 3.2. Effects of elevation and climate

**Figure 3.**
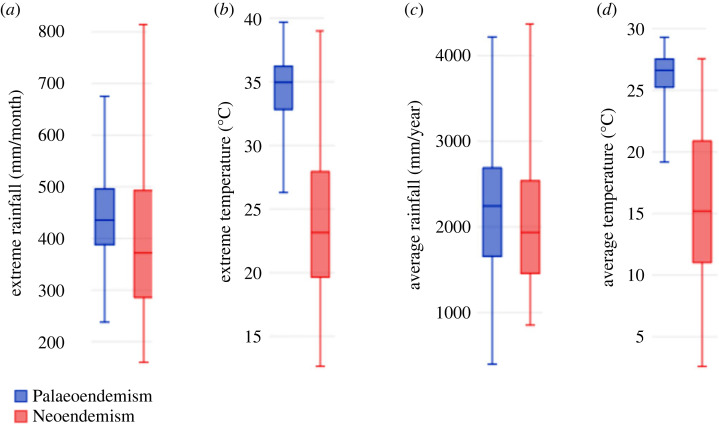
Box and whiskers plot examine the potential relationship between palaeo and neoendemism with the environment. Extreme temperature and rainfall variables were significantly different between evolutionary hotspots (*a,b*). For rainfall (*a*), Student's *t*-test, *t* = 3.13, d.f. = 527.6, ***p < 0.001787. Similarly, for temperature (*b*), the Student's *t-*test showed a result of *t* = 21.5, d.f. = 516.7, ***p < 0. Average rainfall was not significantly different between evolutionary hotspots, Student's *t*-test, *p* < 0.16 (*c*). Average temperature was significantly different between evolutionary hotspots, with the Student's *t*-test producing a result of *t* = 22.6, d.f. = 30.7, ***p < 0 (*d*).

The results of the potential correlation between the areas of palaeoendemism/neoendemism and climate variables support the hypothesis that elevation has a strong level of dependency on climate variables [4], particularly with temperature, either in average or extreme values (figure 3). Interestingly, average precipitation did not show significance, which could be attributed to the complex local patterns of rainfall in Colombia and the limitations of using average data to capture those causal differences.

In summary, the results underscore the pivotal role of elevation and temperature in elucidating the processes underlying the formation of biodiversity cradles and museums, echoing findings presented in the context of the northern Andes [1–6]. Additionally, employing phylogenetic data provides another advantage for biodiversity analysis, as previously demonstrated in the Andes. In this region, cradles and museums of hummingbird biodiversity were distinctly influenced by geography and habitats [7]. However, despite the similarities between the outcomes obtained here and those from hummingbird studies in the northern Andes, a key distinction exists. Unlike the hummingbird case, the old museums of endemic species are not necessarily clustered [7].

Considering this study focuses on flowering plants within a hyperdiverse region, it represents the first comprehensive endeavour to quantify centres of evolutionary diversity, thereby offering novel insights and contributing to our understanding of biodiversity patterns in Colombia. In other tropical countries, such as Africa, centres of palaeoendemism and neoendemism for woody plants have been found overlapping in the mountainous regions of western Africa [6]. This highlights the importance of exploring evolutionary diversity within regional hotspots, as they may exhibit contrasting patterns.

### 3.3. Regions of endemic phylogenetic diversity

**Figure 4.**
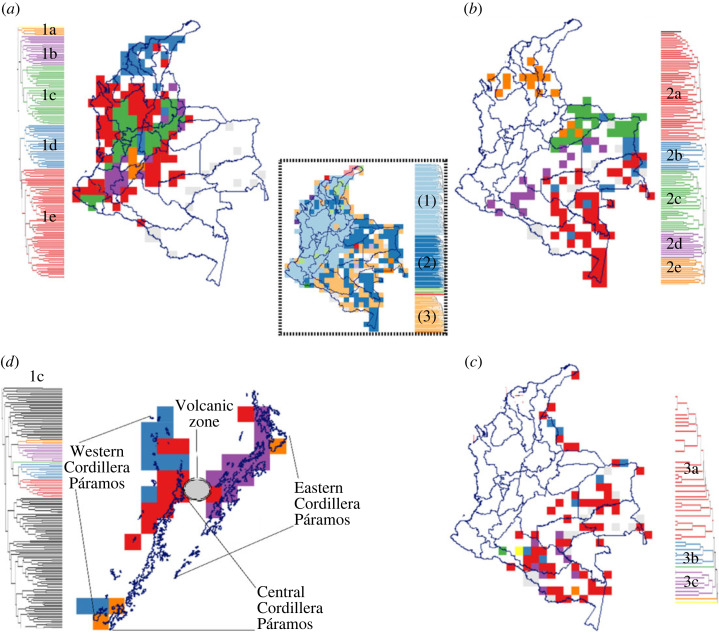
RWT of flowering plants in Colombia. The central map showcases the three primary phylogenetic regions. The five subregions (1a*–*e) constituting region 1 in the western part of the country are depicted in (*a*). Similarly, (*b*) displays the five subregions forming region 2 in the eastern part of the country. Panel (*c*) presents the sub-regions belonging to region 3. Panel (*d*) highlights the Páramo ecosystems, where the three sub-regions contribute to distinctive phylogenetic regions.

To gain a more robust understanding of biodiversity patterns, it is vital to examine not only sites but entire regions. This biogeographic approach offers new perspectives by connecting communities and allowing for the assessment of relationships between regions. Initially, six biogeographic provinces for plants were proposed in Colombia, using the same database as the present study [22]. These provinces were delineated based on species turnover index, without considering evolutionary aspects. However, the results obtained from the application of the RWT in this study reveal previously unidentified evolutionary connections (figure 4).

Based on the results of the RWT, we found that the Andean region stands out as evolutionarily distinct (regions 1a–c in figure 4*a*). This evolutionary distinctiveness supports the idea that cradles of diversity, or centres of neoendemism, are exclusive to the Andes. Interestingly, the northernmost part of the Eastern Cordillera may serve as a connecting point with the lowland provinces of the Caribbean. Within the Andean region, we have identified smaller endemic regions such as the Colombian Massif (region 1b in figure 4*a*) and the Magdalena Dry Depression in the Huila department (region 1a in figure 4*a*). Another closely related group corresponds to the foothill zones surrounding all the mountain ranges (region 1e in figure 4*a*), excluding the dry valleys of the Magdalena and Cauca rivers. This foothills group spans from medium to low elevations and includes the super-humid subgroup of the Darién-Chocó and southern Pacific coastal rainforests. This evolutionary relationship suggests a potential connection between the Amazon and lowlands in the interior of the country, as the eastern slope of the Eastern Cordillera features transitional ecosystems towards the Amazon. Indeed, all these regions exhibit characteristics reminiscent of museums, which further supports the idea that low elevations promote centres of palaeoendemism, even at the regional level. Lastly, the dry Caribbean region appears as an independent subgroup, but still closely related to the foothills group (region 1d in figure 4*a*).

The phylogenetic regions in the Amazon (figure 4*b*,*c*) showed some similarities to the taxonomic regions, but there are notable evolutionary differences that were not previously observed. One such difference is seen in the humid Caribbean region (region 2e in figure 4*b*), which has some degree of evolutionary relatedness with the flooded savannahs of the Orinoco, suggesting shared origins of plants in these geographically separated zones by the Eastern Cordillera. This finding provides new evidence of a possible evolutionary connection between plant communities in the Amazonia and the western part of the country, potentially facilitated by the Serranías corridor between Colombia and Venezuela. As previously demonstrated, the Amazon region is the major source of biodiversity in the Neotropics [32]. Additionally, region 1a in figure 4*a* could have served as another connecting corridor between the Amazon and the country's interior, as the low elevation corridor of Huila shares proximity with the Caquetá foothills region 2d in figure 4*b*. This potential southern connectivity could account for the presence of evolutionary museums in the southernmost valleys of the Magdalena and Cauca rivers. Furthermore, the southern and eastern regions of the Amazon are grouped together (regions 3a–c in figure 4*c*), but they still display transitional zones with multiple elements from various subgroups, suggesting the potential formation of cradle and museum islands in this part of the country.

### 3.4. Páramos as an example of upper mountain cradles

Notably, the Páramo ecosystems stood out as distinct cradle zones, resembling island-like areas, and suggesting the influence of speciation processes [34]. We observed that the Páramo systems align with most evolutionary cradles areas in the high elevations of the Andes. Previous studies focusing on palynology and past climate change have revealed the dynamic nature of Páramo connectivity over the last 1 million years [35].

Based on the results of the RWT, we identified an evolutionary subgroup that specifically represents the Páramos, with distinct clusters for each cordillera (figure 4*d*). The analysis revealed a direct relatedness between the cluster of the Western Cordillera and the cluster of the Central Cordillera, suggesting an evolutionary connection between these regions. This evolutionary connection is probably facilitated through the volcanic zone in the central part of the country. These evolutionary and biogeographic patterns support the flickering theory of Páramos connectivity [35].

The Páramo flickering theory [35], which suggest that Páramo species have shifted to the upper elevations of the Andes over time. Similarly, some inner areas of the Cordilleras, known as Andean plateaus, were identified as part of the evolutionary cradles. For example, the slopes towards mid-elevations were also observed as part of the mountain ranges fitted as cradles areas. This implies that some of the cradle areas may exhibit a combination of range-restricted long and short branches. As previously observed [33], these transitional zones to high elevations probably acted as connectors between humid rainforests and upper mountain forests, potentially facilitating taxa interchange between lowland and highland species during the interglacial Pleistocene period. The findings regarding the elevational distribution of both types of evolutionary hotspots, neo and palaeoendemism, provide support for the altitude-dependent hypothesis [4].

According to the analysis of plant pollen [35], the Páramos (high mountain ecosystems) of the Western Cordillera and Sierra Nevada de Santa Marta are the most isolated in the country and have the lowest level of connectivity among them. By contrast, the Páramos of the Central and Eastern Cordilleras show a closer geographical affinity probably due to their topographic connection. Interestingly, the Páramos of the Central Cordillera experienced the highest degree of fragmentation during the Pleistocene, while the Páramos of the Eastern Cordillera underwent significant movement (up–down shifting on elevation).

### 3.5. Potential biases

There are several aspects that could introduce biases or impact the quality of the results. Weaknesses in the databases can exacerbated these issues, including limited taxonomic diversity, low sampling levels [24], and structural variations in the phylogeny. It is important to know that the results, in taxonomic terms, may not encompass the entire species numbers of the country. Despite this, a significant subset of the flowering plants was captured in this dataset. According to some of the latest biodiversity surveys, there are reports of 24 924 species of vascular plants in Colombia [36]. This subset of species studied here accounts for approximately 15% of the reported vascular flora. At the genus level, Colombia is reported to have a total of 2740 genera of vascular plants [37]. In this dataset, there are 1368 genera, including 19 out of the 25 most diverse genera of vascular plants in Colombia [37]. The most species-diverse genera that were not included in this dataset belong to the Orchidaceae and Cyatheaceae families. At the family level, the studied dataset represents 95% of the major flowering plant groups found in Colombia. Fortunately, spatial phylogenetic analyses can help mitigate taxonomic uncertainties since the phylogeny serves as a taxon-free measure [11].

According to the sampling abundance analyses, one of the flows is that the Amazonian and Orinoco regions have the highest levels of collection gaps. Overall, the sampling abundance per grid is generally around 60% (see electronic supplementary material, figure S2). However, when interpreted at the biogeographic level, this number effectively captures the overall species distribution patterns [22]. Despite the potential weaknesses of the databases, we observe that the evolutionary results reflect the separation of the country's biogeographic regions [38–40].

Finally, biases caused by the type of phylogenetic tree, or its topologies are aspects that could potentially affect interpretations. Although these biases may raise concerns, they might not be all negative. Using the open-source available phylogeny was successful; the results captured and revealed patterns that are biogeographically meaningful. It is also important to note that testing for phylogenetic biases was not the objective of this study. Instead, the assumption was that the quality of the phylogenetic relationships and spatial data was adequate to test the working hypothesis and uncover the cradles and evolutionary museums of flowering plants in Colombia.

## Data Availability

Access to the distributional data used here is granted directly by the supplementary material, and the phylogeny is freely available in [20]. The data are provided in the electronic supplementary material [41].
